# Gestational diabetes mellitus in Greenland: a national study of prevalence and testing efficacy

**DOI:** 10.3402/ijch.v75.32167

**Published:** 2016-08-24

**Authors:** Michael Lynge Pedersen, Jesper Olesen, Marit Eika Jørgensen, Peter Damm

**Affiliations:** 1 Greenland Center of Health Research, Institute of Nursing and Health Science, University of Greenland, Nuuk, Greenland; 2 Queen Ingrid Primary Health Care Center, Nuuk, Greenland; 3 Steno Diabetes Center, Gentofte, Denmark; 4 National Institute of Public Health, Southern Denmark University, Denmark; 5 Center for Pregnant Women with Diabetes, Department of Obstetrics, Rigshospitalet, University Hospital of Copenhagen, Copenhagen, Denmark

**Keywords:** gestational diabetes mellitus, prevalence, screening, Inuit, Indigenous, Greenland

## Abstract

**Background:**

Within the last 20 years, the prevalence of gestational diabetes mellitus (GDM) has been reported to be increasing worldwide in correlation with ethnic and geographic variations. The actual prevalence of GDM throughout all of Greenland remains unknown.

**Objective:**

The aim of this study was to estimate the prevalence of GDM among Greenlanders and non-Greenlanders living in Greenland and to estimate the efficacy of testing for GDM.

**Design:**

This study was performed as an observational, cross-sectional study including all women with permanent address in Greenland who had given birth to a singleton during 2014. The prevalence of GDM was calculated as the proportion of all pregnant women tested with a 75-g 2-h glucose tolerance test who had a 2-h capillary whole-blood glucose value of 8.5 mmol/l or above. Testing efficacy was calculated as the proportion of women who fulfilled the testing criteria who were actually tested in Greenland in 2014.

**Results:**

A total of 794 women (727 Greenlanders and 67 non-Greenlanders) were included in the study. The prevalence of GDM among tested women was 3.3% (confidence interval, CI: 0.9–5.6) among Greenlanders and 12.5% (CI: 0–25.7) among non-Greenlanders, corresponding, respectively, to 1.0% (CI: 0.3–1.3) and 4.5% (CI: 0–9.4) of all singleton pregnancies in Greenland in 2014. The overall testing efficacy was 69.0% among all eligible residents of Greenland and 85.1% among eligible residents in the capital city, Nuuk.

**Conclusion:**

In conclusion, the prevalence of GDM seems quite low in Greenland. Although diagnostic testing activity has improved within the last 6 years, still around one-third of all pregnant women in all Greenland fulfilling the testing criteria were not tested. Universal testing for GDM may be needed to improve testing of GDM in Greenland.

Gestational diabetes mellitus (GDM) is defined as hyperglycaemia diagnosed during pregnancy (1). GDM is a serious condition associated with maternal and offspring complications like preeclampsia, shoulder dystocia, caesarean delivery, macrosomia, neonatal hypoglycaemia and hyperbilirubinaemia (2–5). Women diagnosed with GDM have an increased risk at around 50% of developing diabetes later in life (6,7). Also, offspring of women with GDM have increased risk of developing obesity and diabetes as children or in adulthood (7–11). The prevalence of GDM has been reported to be increasing worldwide within the last 20 years (12,13), and the offspring risk of developing obesity and diabetes may contribute to the worldwide epidemic of these conditions (12). Both ethnic and geographic variations related to the prevalence of GDM have been reported (13). Prevalence estimates around 2–6% have been reported in Europe with a trend towards higher prevalence in southern Europe (13). Observations from Australia and the United States report a higher prevalence of 5–8% among Asians, Hispanics, Africans (12,13) and among Yupik Inuit (14). Only one study has been performed investigating GDM among Greenlanders in Greenland where the prevalence of diabetes and obesity has been increasing over the last two decades (15–17). A low prevalence of GDM was previously reported; however, that study was relatively small, and only inhabitants of the largest city of Greenland, Nuuk, were included in the study (15). Thus, the nationwide prevalence of GDM in Greenland remains unknown. Identifying women with GDM is important since appropriate glycaemic control during pregnancy can reduce adverse perinatal outcomes (18,19). GDM is most often asymptomatic and must be diagnosed by testing with an oral glucose tolerance test (OGTT) (13,20). Testing practices for GDM are inconsistent worldwide, even across European countries (13). Practices range from universal testing of all pregnant women to testing on a case-by-case basis according to clinician or patient decisions (13). In Greenland, testing for GDM is performed in high-risk cases following the same criteria used in Denmark (21). The testing efficacy in the 2008 study in Nuuk was reported as suboptimal (15). However, the actual testing efficacy for all of Greenland remains unknown. Thus, the aim of this study was to estimate the prevalence of GDM – as well as the testing efficacy – on a population-based scale including all pregnant women who gave birth in Greenland in 2014.

## Material and methods

This study was performed as an observational, cross-sectional study based on the review of register data and medical records.

Greenland is the biggest island in the world covering approximately 2 million km^2^. The 56,000 inhabitants live in 16 towns and approximately 60 settlements along the coastline. Healthcare is delivered by one united public healthcare system, free of charge to anyone with a permanent address in Greenland. Around half of all birth deliveries in Greenland take place in the capital, Nuuk, where the only obstetric department in Greenland is located. Pregnant women experiencing complications during pregnancy are referred to Nuuk. The remaining births take place locally at small hospitals in smaller towns and settlements. Prenatal care including first prenatal care is performed by midwives in Nuuk and in some of the larger towns in Greenland. In settlements and towns without midwives, prenatal care is performed by other healthcare professionals, by visiting midwives or the pregnant women visiting towns with midwives. At all settings prenatal care and medical information are documented in a systemized perinatal record. All births in Greenland are reported to the Chief Medical Officer (CMO) in Greenland.

The testing criteria and use of 2-h blood glucose values follow the guidelines published by the Danish Society of Obstetrics and Gynaecology in 2014 (22). The choice to test for GDM is based on any one of the following criteria: overweight (pre-pregnant body mass index (BMI) ≥27 kg/m^2^), family history of diabetes among first-degree relatives or grandparents, previous delivery of an infant with a birth weight ≥4,500 g and GDM during earlier pregnancies. Finally, all pregnant women with glucosuria are tested. Testing is performed using a 75 g OGTT at 28 gestational weeks. In addition, an OGTT is performed at 18 gestational weeks if more than one risk factor is present or if GDM was observed in a former pregnancy. Diagnosis of GDM in Greenland is based on capillary blood examined 2 h after the administration of glucose.

All women who had given birth in Greenland in 2014 were identified from the register held by the CMO. Information about maternal age, country of birth, parity, gestational age (GA) at delivery, mode of delivery, offspring sex, and birth weight and length was available for all the women. In addition, information about maternal weight, height, the results of examination of urine for glucose, former pregnancies, family history of diabetes, and smoking and alcohol use was obtained from the perinatal records. Results from the OGTT were collected from information recorded on the laboratory cards used in Greenland. Women born in Greenland were considered as Greenlanders, whereas women born outside Greenland were considered as non-Greenlanders. Only women with a singleton pregnancy were included in this study. Women with pre-gestational diabetes and women treated with oral steroids were excluded from the study.

The OGTT was performed in fasting, pregnant women with the administration of 75 g pure glucose diluted in water. Capillary blood was examined 2 h after the administration for glucose using a portable Hemocue^®^ Glucose 201 + System (AB, Angelholm, Sweden), which was calibrated weekly. GDM was defined according to the International Association of Diabetes and Pregnancy Groups (IADPSG) recommendations on the Diagnosis and Classification of Hyperglycaemia in Pregnancy using only a 2-h (venous plasma) value of 8.5 mmol/l or above (20). Thus, all women with a 2-h capillary whole-blood glucose measurement of 8.5 mmol/l or above were considered having GDM though assuming an acceptable agreement between glucose measured in capillary whole blood and venous plasma (23).

BMI was based on the subject's self-reported weight and height before pregnancy. The women were considered to be smokers if they reported any tobacco smoking at the first prenatal visit and were considered to be alcohol users if any alcohol use was reported at the first prenatal visit. Delivery was considered vaginal unless a caesarean section had been performed. Women without a history of former delivery of a child at GA at or above 28 weeks were classified as nulliparous. GA was based on the subject's self-reported last menstrual period (LMP). In cases where LMP was unknown and in cases where an ultrasound-based GA calculation deviated 2 weeks or more from the LPM-based GA, the ultrasound-based GA was used.

The prevalence of GDM was calculated as the proportion of women with GDM among women screened with OGTT, and all women giving birth in Greenland during 2014 were included in the study. The efficacy in the testing procedure was determined as the proportion of women meeting the testing criteria (see above) that were actually tested with at least one OGTT. Ninety-five percent confidence intervals (CI) were used in the study. Variables were described using medians and interquartile range. Medians were compared using Mann–Whitney U test. Logistic regression was used to study the association between Greenlanders and non-Greenlanders and GDM, adjusting for age. Frequencies were compared using Fischer's exact test. A two-sided p-value below 0.05 was used as the level of significance. Statistical analyses were performed using SPSS statistical software, version 23.0 (Norusis; SPSS Inc., Chicago, IL).

The study was approved by The Ethics Committee for Medical Research in Greenland and the Agency of Health and Prevention in Greenland.

## Results

In total, 794 women (727 Greenlanders and 67 non-Greenlanders) giving birth to a singleton in Greenland during 2014 were identified from the CMO's register and included in the study. None of the women gave birth more than once during the study period. No cases were excluded due to pre-existing diabetes or treatment with oral steroids prior to pregnancy. Perinatal records with additional information about the pregnancy were identified in 620 cases (78%), while the remaining 174 perinatal journals could not be located.

Basic characteristics for the women are shown in Table I. Greenlanders were younger, shorter, more frequent smokers, had higher parity, gave birth at a lower GA and less often by caesarean sections. Risk factors for GDM, testing proportion and prevalence of GDM are shown in Table II. In total, 49.4% (303/613) of the women presented at least one of the five risk factors for GDM with BMI above 27 kg/cm^2^ as the most prevalent risk factor observed in one-third of all women. The testing efficacy among women with at least one risk factor was 69.0% (209/303) for all pregnant women in Greenland and 85.1% for Nuuk residents. The testing efficacy among Greenlanders was 67.6% (188/278), which was lower than the 84.0% (21/25) among non-Greenlanders (p=0.047). In total, 30.1% (239/794) of all the women were tested during pregnancy. The crude prevalence of GDM among tested women was 4.2% (CI: 1.6–6.7). The prevalence among tested Greenlanders was 3.3% (CI: 0.9–5.6) and among tested non-Greenlanders was 12.5% (CI: 0–25.7) (p=0.02) corresponding to a prevalence of 1.0% (CI: 0.3–1.7) and 4.5% (CI: 0–9.4) (p=0.019) of all women included in the study. However, when adjusting for age by logistic regression, no difference was found between Greenlanders and non-Greenlanders (Table II).

**Table I:** Basic characteristics of pregnant women in Greenland 2014

Characteristics and variables	Greenlanders Median (IQR) N=727	Non-Greenlanders Median (IQR) N=67	p
Maternal age (years)	26 (8.0)	31 (6.0)	**<0.001**
Height (cm)^a^	161 (9.0)	167 (12.0)	**0.014**
Weight before pregnancy (kg)^b^	65 (19.3)	64 (24.5)	0.784
BMI (kg/m^2^)^c^	24.5 (6.9)	23.7 (5.5)	0.696
Gestational age at delivery (days)	274 (12.0)	277 (13.3)	**0.008**
Birth weight (g)	3,575 (725)	3,495 (770)	0.798
Birth length (cm)	51 (3.0)	52 (3.0)	0.173
Nulliparous (%)	39.9 (289/724)	53.7 (36/67)	**0.037**
Vaginal delivery (%)	93.5 (680/727)	77.6 (52/67)	**<0.001**
Smoking during pregnancy (%)	55.3 (316/571)	23.3 (14/60)	**<0.001**
Alcohol during pregnancy (%)	3.0 (17/570)	1.7 (1/60)	>0.999
Male offspring (%)	51.4 (374/727)	46.3 (31/67)	0.445

a N=561

b N=565

c N=559, p-value <0.05 in bold, inter quartiles range (IQR).

**Table II:** Risk factors, testing proportions and prevalence of gestational diabetes (GDM) among women included in the study

Risk factor	Greenlanders (%) (n/N)	Non-Greenlanders (%) (n/N)	p	All women (%) (n/N)	Proportion tested (%)
Diabetes in former pregnancy	0.2 (1/566)	0 (0/60)	>0.999	0.2 (1/626)	100 (1/1)
Family history of diabetes	14.1 (80/566)	23.7 (14/59)	0.056	15.0 (94/625)	67.0 (63/94)
Previous delivery of an infant with a birth weight ≥4,500 g	3.9 (22/563)	1.7 (1/59)	0.714	3.7 (23/622)	87.0 (20/23)
BMI ≥ 27 kg/m^2^	35.3 (196/555)	20.0 (12/59)	**0.021**	33.9 (208/614)	71.6 (149/208)
Glycosuria	9.3 (56/603)	3.2 (1/31)	0.348	9.0 (57/634)	33.3 (19/57)
At least one risk factor	50.1 (278/555)	43.1 (25/58)	0.336	49.4 (303/613)	69.0 (209/303)
At least one risk factor Nuuk residents	45.1 (74/164)	51.3 (20/39)	0.592	46.3 (94/203)	85.1 (80/94)
OGTT performed all women	29.6 (215/727)	35.8 (24/67)	0.330	30.1 (239/794)	30.1 (239/794)
GDM all women (95% CI) and (n/N)	1.0 (0.3–1.7) (7/727)	4.5 (0–9.4) (3/67)	0.121*	1.3 (0.5–2.0) (10/794)	–
GDM tested women (95% CI) and (n/N)	3.3 (0.9–5.6) (7/215)	12.5 (0–25.7) (3/24)	0.161*	4.2(1.6–6.7) (10/239)	–

* Age-adjusted, p-value <0.05 in bold, gestational diabetes mellitus (GDM).

## Discussion

The overall prevalence of GDM among all tested women who gave birth to a singleton in 2014 in Greenland was estimated to be 4.2% (CI: 1.6–6.7). No difference in prevalence of GDM was observed between Greenlanders and non-Greenlanders when adjusting for age. Around one-third of all women fulfilled the testing criteria for GDM used in Greenland, and of those, 69.0% were actually tested. The highest testing efficacy (85.1%) was achieved among Nuuk residents.

A major strength of this study is that it is the first study to include all pregnancies in Greenland and only the second study on GDM performed in Greenland. However, limitations exist and the results must be interpreted with some reservations. The true overall prevalence of GDM among all pregnancies in Greenland is most likely lower than the 4.2% reported among all screened women because these women represent women with at least one risk factor for GDM. On the contrary, the prevalence among all women must be higher than the 1.3% reported in this study because not all women with a least one risk factor were screened. In addition, GDM may also be found among women without any known risk factors. Thus, changing testing strategy among Aboriginals in Australia from selective risk-factor-based screening to universal screening resulted in an increase of 40% in prevalence of GDM, underlining that GDM can occur among women without risk factors (24). A recent study identified a common mutation in *TBC1D4* causing muscle-tissue-specific insulin resistance and postprandial hyperglycaemia (25). This variant accounts for around 15% of all diabetes among Greenlanders and is not associated with usual diabetes risk factors. This mutation in *TBC1D4* is most likely related to high risk of GDM (25). Thus, also pregnant women without other known risk factors for GDM may have the mutation and, thus, actually be at high risk of GDM. However, with a risk-factor-based test strategy, these women will not be offered an OGTT, and consequently, cases of GDM may remain undiagnosed.

Another limitation is that not all the perinatal records could be located, leading consequently to incomplete information about risk factors for some of the pregnancies. Still, 78% of the perinatal records were included, and we have no reason to believe that the missing perinatal records represent a systematic selection bias. In addition, the estimated prevalence of GDM was not affected by the missing perinatal records because information about OGTT was available for all women in the laboratory system. A total of 9.3 and 3.2% of Greenlandic and non-Greenlandic women, respectively, had glycosuria. However, only one-third of these women underwent testing, suggesting that GDM is underestimated for this risk factor criterion. Also, in this study only 2-h capillary whole-blood glucose values could be included since no laboratory in Greenland measures glucose concentrations in venous plasma. Thus, it is likely that some women with GDM according to IADPSG (20) thresholds for fasting or 1-h plasma glucose tests have not been diagnosed by the 2-h capillary whole-blood glucose concentrations alone. Consequently, the prevalence reported in this study might be underestimated and GDM under-diagnosed in Greenland because diagnostic testing is not universal, but risk factor based, and does not include a fasting or 1-h glucose value test.

The prevalence reported in this study is similar to the prevalence (4.3%) reported among Greenlandic women who gave birth in Nuuk during 2008 (15). The prevalence of GDM in Greenland seems to be comparable internationally with northern Europe with its prevalence at 2–4% (13). Also, the prevalence of GDM among Greenlanders seems comparable with or even lower than prevalence among non-Hispanic white people in the United States (2.9–6.9%) and non-Aboriginal people in Australia (3.1–7.5%) (12). Higher prevalence has been reported among Native Americans, Asians, Hispanics and African-Americans living in the United States than among non-Hispanic white people and among Aboriginals compared with non-Aboriginals in Australia (12,13). This is in contrast to this study where the lowest crude prevalence was observed among the Greenlanders. Yet, no difference was observed when adjusting for age.

A country-wide test efficacy in Greenland of 69.0% reported in this study, and the 85.1% in Nuuk is much higher than reported in Nuuk in 2008 (54%). This may reflect the increased awareness of diabetes in Greenland where the prevalence of diagnosed diabetes has also been steadily increasing within the last decade (16,17). The higher test efficacy in Nuuk most likely reflects that the only obstetrics department in Greenland is also situated in Nuuk. The minor, geographically isolated healthcare clinics without specialists outside of Nuuk are challenged in the delivery of all kinds of primary healthcare including the care of patients normally dealt with by specialists. Also, lack of healthcare professionals and the employment of short-term, temporary workers challenge the continuity in delivering healthcare locally. Routine, universal testing for GDM might help increasing the test efficacy, but this decision has to be balanced with the difficulty of performing the cumbersome OGTT and the relatively low prevalence of GDM among Greenlanders reported in this study, which thus might not warrant the effort.

In conclusion, the prevalence of GDM seems quite low in Greenland. Although diagnostic testing activity has improved within the last 6 years, around one-third of all pregnant women in all Greenland fulfilling the testing criteria were still not tested. Universal testing for GDM may be needed to improve testing of GDM in Greenland.

## Key finding

The prevalence of GDM seems low among Greenlanders in contrast to reports of results from testing many other Indigenous and non-white populations. Despite increased testing for GDM in Greenland, compared to 2008, GDM is most likely still under-diagnosed in Greenland.
